# Cathodal tDCS Over Motor Cortex Does Not Improve Tourette Syndrome: Lessons Learned From a Case Series

**DOI:** 10.3389/fnbeh.2018.00194

**Published:** 2018-08-24

**Authors:** Nora Behler, Bianka Leitner, Eva Mezger, Elif Weidinger, Richard Musil, Bernhard Blum, Beatrice Kirsch, Linda Wulf, Lisa Löhrs, Christine Winter, Frank Padberg, Ulrich Palm

**Affiliations:** ^1^Department of Psychiatry and Psychotherapy, Ludwig-Maximilian University, Klinikum der Universität München, Munich, Germany; ^2^Department of Neurology, Ludwig-Maximilian University, Klinikum der Universität München, Munich, Germany; ^3^neuroCare Group, Munich, Germany; ^4^Department of Psychiatry and Psychotherapy, Charité Universitätsmedizin Berlin, Berlin, Germany

**Keywords:** tDCS, transcranial direct current stimulation, GTS, tourette syndrome, supplementary motor areas, OCD, obsessive compulsive disorder

## Abstract

**Introduction**: Current pathophysiological hypotheses of Gilles de la Tourette Syndrome (GTS) refer to temporally abnormal neuronal activation in cortico-striato-thalamo-cortical (CSTC) networks. Modifying cortical activity by non-invasive brain-stimulation appears to be a new treatment option in GTS.

**Background**: Previous studies suggested therapeutic effects of cathodal transcranial direct current stimulation (tDCS) to pre-supplementary motor areas (SMA), however, treatment modalities concerning electrode placement, current intensity and stimulation-rate have not been systematically explored. Aim of this study was to assess efficacy of an alternative stimulation regime on GTS symptoms in a pilot study. To test a treatment protocol with tDCS twice a day, we administered 10 sessions over 5 days of bilateral cathodal tDCS (30 min, 2 mA) over the pre-SMA in three patients with severe GTS. Tic severity as well as obsessive-compulsive (OC) symptoms and affective scales were rated before and after tDCS treatment.

**Discussion**: Only one out of three patients showed a 34.5% reduction in tic severity. The two other patients showed an increase in tic severity. All patients showed a mild increase in positive affect and a reduction in negative affect, OC symptom changes were heterogeneous. Our results do not support earlier findings of extensive therapeutic effects of cathodal tDCS on tics in patients with GTS and show that prediction of stimulation effects on a targeted brain area remains inaccurate.

**Concluding Remarks**: Future research will have to focus on the determination of most effective stimulation modes regarding site, polarity and frequency of tDCS in GTS patients.

## Introduction

Gilles de la Tourette syndrome (GTS) is a neuropsychiatric disorder with chronic motor and vocal tics, manifesting in the course of childhood and early adolescence. Tics are usually preceded by premonitory urges and can be suppressed at will. The disorder is self-limiting in at least 44% of all cases and symptoms tend to significantly decrease in early adulthood. Still there are 22% of adult patients retaining clinically significant symptoms despite adequate medication (Leckman et al., 1998; Burd et al., 2001; Pappert et al., 2003; Evans et al., 2016). Approved pharmacological treatment consists of alpha-2-adreno-receptor-blockers, and distinct antipsychotics. Both groups of medications are associated with mild to severe side effects including sedation, cardiovascular dysregulation, extrapyramidal motor symptoms (EPMS), sexual dysfunction, weight gain or cardiac risks that are scarcely tolerated by patients. Moreover, depression and anxiety are common co-morbid disorders in adult GTS patients (Evans et al., 2016), and there is an additional need for specific treatment in these domains. Psychotherapeutic treatment such as habit reversal training or exposure with response prevention require sufficient adherence while also yielding only incomplete remission rates (Capriotti et al., 2014). Tic manifestation in GTS is usually referred to an organic etiology. Functional neuroimaging has revealed that tic symptoms originate from a dysregulation of cortico-striato-thalamo-cortical (CSTC) networks. Results show a down regulation of movement inhibition in caudate and anterior cingulate cortex and an hyperactivation of motor pathways in putamen, pallidum and substania nigra, as well as in cortical regions of the sensorimotor cortex with cortical hyperactivation in pre-motor regions preceding basal ganglia activation (Wang et al., 2011). Therefore, modulation of basal ganglia function by deep brain stimulation (DBS) might offer a third track of treatment besides psychopharmacology and psychotherapy. DBS has shown promising results in GTS while resulting in high operative risk and diverse side effects, restricting treatment to a severely affected group of adult patients (Visser-Vandewalle et al., 2014; Schrock et al., 2015). In summary, treatment alternatives are sparse and results are dissatisfying due to lack in treatment response or extensive side effects. In view of the success in DBS and the implication of cortical dysregulation, non-invasive brain stimulation (NIBS) appears a viable choice without the risk of invasive treatments. Repetitive transcranial magnetic stimulation (rTMS), inducing an electric current in cortical regions via a pulsed magnetic field, has been shown to be efficient in tic reduction when applied at 1 Hz over the supplementary motor area (SMA; Mantovani et al., 2006). In children, positive results lasted at least 6 months (Le et al., 2013). However these studies were open label without sham control. Landeros-Weisenberger et al. (2015) presented the first sham controlled double-blind rTMS study in patients with severe GTS and could not show a significant difference in tic improvement after active rTMS compared to sham rTMS. Thus, results of rTMS in the treatment of GTS are controversial so far (Kious et al., 2016; Pedroarena-Leal and Ruge, 2017). Another emerging NIBS technique, transcranial direct current stimulation (tDCS) has been shown to modulate motor cortex excitability (Nitsche and Paulus, 2001) and frontal network activity (Keeser et al., 2011a). The modulation of large scale brain networks close to the stimulation site and in remote areas could serve as a surrogate for a hypothesized influence of tDCS on activation in basal ganglia. Altering activity in whole-brain networks by stimulation of specific nodes has been proposed as a mode of treatment in GTS, while the appropriate dosage and targets remain to be established (Pedroarena-Leal and Ruge, 2017). tDCS is considered safe, with very limited side effects, such as itching of the skin, light headaches or dizziness (Nitsche et al., 2003; Bikson et al., 2016). Commonly, daily treatment is performed in acute phases of disease and intermittent and even home treatment are being explored in chronic therapeutic settings (Charvet et al., 2015; Palm et al., 2018) and in phases of remission, treatment-free periods can be elongated according to individual patient needs. These options appear especially appealing in a usually young and mentally unimpaired patient group such as GTS patients.

In tDCS, polarity of stimulation over the affected cortical region is a determinant of treatment effects. Cathodal stimulation is thought to have inhibiting effects, while anodal stimulation inversely has increasing effects on cortical excitability (Nitsche and Paulus, 2001). As functional magnetic resonance imaging (fMRI) revealed increased activity preceding tic manifestation in cortical pre-motor and motor regions (Wang et al., 2011), cathodal tDCS over these areas may reduce excitability in these regions. Previous findings in single GTS patients suggested therapeutic effects of cathodal tDCS of pre-SMA both via monolateral treatment of the most affected side or bilateral treatment, with extracephalic anodal reference electrodes, respectively (Mrakic-Sposta et al., 2008; Carvalho et al., 2015). Though randomized controlled clinical trials (RCT) investigating tDCS in GTS are lacking, further data together with an RCT protocol have been published recently (Eapen et al., 2017). There, patients will be treated with 18 sessions of 1.4 mA tDCS with the cathode positioned over SMA area and the reference electrode over the right deltoid muscle. At least one of the two pilot patients showed relevant improvement of tic severity during treatment period. As optimal tDCS parameters have not been identified to date, we investigated twice-daily sessions of bilateral cathodal tDCS over the pre-SMA for 5 days in three patients with GTS. This approach aims to assess a different stimulation regime from previous studies to further knowledge on stimulation effects in patients with this condition.

## Materials and Methods

Three GTS patients from the Department of Psychiatry, University of Munich, underwent twice-daily tDCS treatment for 5 days. All patients gave their written informed consent for an individual treatment attempt by use of tDCS as an experimental compassionate use and agreed on their data being anonymously published after completion of the treatment. Pre-existing pharmacological treatment was not altered before or during tDCS treatment and, if receiving psychopharmacologic drugs, patients had a stable dosage for at least 3 weeks without change of motor symptoms.

### Transcranial Direct Current Stimulation

**Figure 1:**
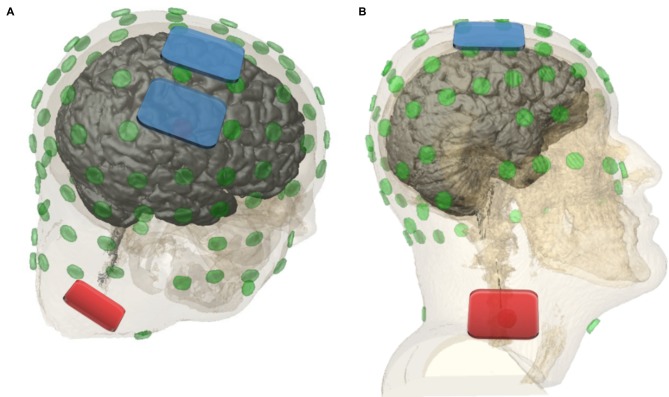
Dorsal lateral view **(A)** and lateral view **(B**; Soterix Medical HD-Explore™). Cathodal electrodes (7 × 5 cm = 35 cm^2^) were placed bilaterally over (pre-)motor cortical areas (C3, C4) with 1 cm space in between. Anodal electrodes were placed ipsilateral over the sternocleidomastoid muscle. Current strength of 2 mA was applied for 30 min.

Two milliampere tDCS was applied twice a day for 30 min (in total 10 stimulations in 5 days) following previous findings on safety of repeated twice-daily tDCS in depressive disorders (Palm et al., 2015). Both tDCS sessions were performed in the morning to avoid further circadian influences and were separated by an interval of approximately 3 h (e.g., 8:00, 11:00). Two independent stimulators were used parallel to stimulate both hemispheres. Cathodal stimulation was performed using rectangular 7 × 5 cm = 35 cm^2^ saline soaked sponge electrodes, placed bilaterally and longitudinally over motor areas of the cortex (C3, C4, according to 10-20 EEG system) with 1 cm space between them (Figure 1). With this montage, SMA and pre-SMA areas were also covered. Anodal electrodes were each fixed with adhesive tape ipsilateral over the sternocleidomastoid muscle (extracephalic electrodes). Direct current was applied with an eldith^®^ DC-stimulator (neuroConn, Ilmenau, Germany). For better tolerance, stimulation was ramped up and ramped down over 15 s.

### Measurements

Before and after tDCS series, the following questionnaires were administered: Yale Global Tic Severity Scale (YGTSS; Leckman et al., 1989; Storch et al., 2005) to quantify tic severity, Yale-Brown Obsessive Compulsive Scale (Y-BOCS; Goodman et al., 1989) to assess obsessive and compulsive behavior and the Positive and Negative Affect Schedule (PANAS; Krohne et al., 1996) to evaluate affective symptoms. Tic frequency was assessed by counting tics during a 3 min interval while sitting at rest in a quiet room. To evaluate the effect of tDCS, percentage changes of data obtained at day five as compared to results at baseline were calculated.

## Patient Characteristics and Clinical History

The first patient (P1), a 55 year old male, presented with simple motor and simple vocal tics (age at onset: 9/10 years) as well as obsessive compulsive disorder (OCD). Motor tics included grimacing, grinding of teeth, tensing of shoulder and nuchal muscles, increasing during times of rest and stressful situations. Vocal tics included loud exclamations, sniffing, harrumphing or grunting. Tic frequency was low. OCD symptoms included compulsive checking and writing. Current medication was limited to antihypertensive medication (candesartan 8 mg/day), previous treatment with aripiprazole showed slight reduction in tic frequency with unacceptable side effects of extensive weight gain and was discontinued 4 years ago. Psychotherapeutic treatment was limited to treatment for OCD symptoms several months before undergoing tDCS.

The second patient (P2), a 20 year old male, presented with simple motor and simple vocal tics (age at onset: 16 years). P2 did not show any OCD symptoms. Motor tics included striking out of both arms (with ensuing self-harming effects), jerking of the head and facial muscles. Vocal tics included barking and uttering of syllables. Current treatment consisted of atypical antipsychotics (tiapride 600 mg/day, olanzapine 15 mg/day). Previous treatment also comprised atypical antipsychotics (risperidone, amisulpride, aripiprazole, olanzapine and quetiapine). The patient showed a history of substance-induced psychotic symptoms (tetrahydrocannabinol and “spice”) without current use of illegal substances and was abstinent since 3 years. He also exhibited dissociative seizures during youth that have been treated successfully by an alternative practitioner by eye movement desensitization and reprocessing (EMDR) and hypnosis.

The third patient (P3), an 18 year old female, presented with frequent simple and complex motor as well as vocal tics (age at onset: 14 years). She was also suffering from OCD symptoms. Motor tics included hitting own thorax and pelvis with her fists, flipping, grimacing, jerking of the head, shoulders and hands, gesturing (Russian roulette), saluting and locking her feet while walking. Vocal tics included harrumphing, whistling, caterwauling, uttering syllables, words, limited sentences and echolalia. Tic-free sequences were short (max. 1 min), urges to perform tics were rated very high by the patient. OCD symptoms included compulsive counting, repeating, checking and arranging/collocating. Current treatment consisted of risperidone 6 mg/day and biperiden 4 mg/day in a stable dose for 3 weeks. Previous treatment included antidepressants and atypical antipsychotics (fluoxetine 30 mg/day, aripiprazole 10 mg/day, tiapride 600 mg/day, and quetiapine in unknown dosage) and was discontinued more than 1 year before due to symptom-alleviation (fluoxetine) or lack in positive therapeutic outcome or inacceptable side effects (aripiprazole, tiapride, quetiapine).

## Results

All patients completed twice daily sessions of tDCS for 5 days and stimulations were generally well tolerated. Side effects were mild skin irritation in P1 due to adhesive tape over the electrode, mild headache and metallic taste during stimulation in P2, none in P3.

**Figure 2:**
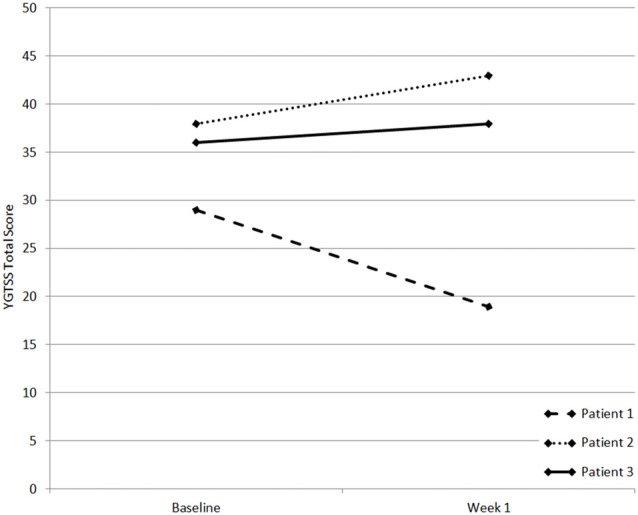
Yale Global Tic Severity Scale (YGTSS) scores of patients 1–3. x-axis shows measuring time points, y-axis shows YGTSS score.

Prior to stimulation, global tic severity scores (YGTSS), not including general impairment scores, were rated at 29, 38 and 36 points for patients 1, 2 and 3, respectively. After tDCS for 5 days, tic-severity decreased in patient 1 by 10 points to finally 19 points. Both patients 2 and 3 showed an increase in tic-severity by 5 points to 43 points (P2) and 2 points to 38 points (P3, Figure 2). General impairment scores as measured by the YGTSS did not differ in comparison between pre and post tDCS measurements. P1 showed impairment of 10 points, P2 30 points and P3 50 points.

OCD symptoms were present only in patients 1 and 3, rated at 18 and 10 points, respectively. Patient 1 reported a reduction of 83.3% in OCD symptoms following stimulation. Patient 3 showed an increase of 20% in OCD symptoms.

Affective symptoms were self-rated and categorized into PANAS negative and positive subscores. All three patients expressed a decrease in negative and an increase in positive mood following stimulation. Most notable changes occurred in patient 2 with an increase of positive affect of 30.8% and a decrease of negative affect of 20%.

The 3 min tic-count showed high inter-individual variability, patients 1 and 2 experienced 11 and 19 tics in 3 min, whereas patient 3 experienced 218 tics in 3 min. Surprisingly, contrary to the reduction in tic severity observed in patient 1, tic-counts showed an increase in all three subjects, following stimulation (increases by 18.2% in patient 1, by 200% in patient 2 and by 70.2% in patient 3). Clinical data is reported in Table 1.

**Table 1:** Clinical rating before (pre) and after (post) transcranial direct current stimulation (tDCS) treatment for 5 days.

Tests	Patient 1			Patient 2			Patient 3		
	pre	post	Δ [%]	pre	post	Δ [%]	pre	post	Δ [%]
YGTSS	29	19	−34.5	38	43	13.2	36	38	5.6
YBOCS	18	3	−83.3	0	0	0.0	10	12	20.0
PANAS neg.	17	16	−5.9	15	12	−20.0	23	19	−17.4
PANAS pos.	34	35	2.9	26	34	30.8	26	27	3.8
tic count (3 min)	11	13	18.2	19	57	200.0	218	371	70.2

## Discussion

Here, we report three cases of GTS patients undergoing bilateral cathodal tDCS of SMA and pre-SMA areas. This intervention did not decrease tic severity in two out of three patients. Only one patient reported a reduction of tic severity, but even more marked of OCD symptoms. In contrast, an increase in tic severity was observed in the other two subjects. All subjects showed an increase in 3 min tic count and a decrease in negative emotions following stimulation as well as a slight increase in positive affect. Thus, the current tDCS protocol over SMA and pre-SMA areas was not successful in treating GTS core symptoms in contrast to previous studies (Mrakic-Sposta et al., 2008; Carvalho et al., 2015; Eapen et al., 2017).

There are clear limitations of our approach: first, the problem of natural waxing and waning of tics in GTS which is adding to the issue of the very small sample size of our observation. Second, the heterogeneous clinical characteristics of our patients and the variance of co-medication (Brunoni et al., 2012; Nitsche et al., 2012) may impact on stimulation efficacy, but also reflect a disorder with frequent and typical side diagnoses and insufficient efficacy of singular pharmacotherapy. Of note, regarding GTS epidemiology, P2 and P3 showed an untypically late onset of tics at the age of 16 and 14 years respectively and did not show any therapeutic efficacy, whereas P1 had a more typical tic onset at 9 years of age and showed a decrease in tic severity following tDCS. This difference in age at onset might constitute an indicator for efficacy of tDCS in GTS, eventually referring to differences in pathology and etiology. A major limitation is the lack of a sham tDCS condition, however, the application of tDCS was for compassionate use and not part of a study protocol. Thus, all effects observed may be due to placebo or nocebo effects of stimulation.

Concerning stimulation parameters, i.e., current strength, duration and number of sessions per day, we deviated from previously reported cases in GTS and this also might have contributed to our negative result. In this respect our case series was rather designed to empirically explore a new stimulation regime than to reproduce and validate earlier findings, since to this end, larger controlled trials would be needed rather than small case series. In comparison, Carvalho et al. (2015) applied a more focal stimulation, using smaller surface electrodes that presumably resulted in a more specific stimulation of the pre-SMA. Similarly, Mrakic-Sposta et al. (2008) used smaller electrodes but applied current more generally over the motor cortex, and Eapen et al. (2017) applied electrodes with a size of 25 cm^2^ over the anterior portion of the SMA. Although supported by evidence of the proposed parameters in the treatment of major depression (Palm et al., 2016) and taking into account a lack in large controlled studies on effective stimulation parameters in GTS, our less focal approach may not have been an optimal mode of stimulation. However positioning of reference electrodes is the same across cases with extracephalic placement over right deltoid muscle (Mrakic-Sposta et al., 2008; Carvalho et al., 2015; Eapen et al., 2017) whereas we used an ipsilateral montage on sternocleidomastoid muscles.

A further difference was the use of 2 mA intensity here and in Mrakic-Sposta et al.’s (2008) study instead of 1.4 mA (Carvalho et al., 2015; Eapen et al., 2017). This intensity has been selected based on our experimental studies of prefrontal tDCS in humans (Keeser et al., 2011a,b) and treatment parameter for major depression (Brunoni et al., 2013). These empirically established parameters were adopted from recent findings in the treatment of major depression where a tDCS relationship between total dosage and efficacy of stimulation was found (Brunoni et al., 2016), but these results are restricted to anodal prefrontal stimulation. Therefore, our cathodal tDCS protocol was rather comparable to the protocol applied in a randomized controlled trial in OCD by D’Urso et al. (2016). Interestingly, the largest and clinically meaningful effect was not found on GTS, but on OCD symptoms, i.e., a reduction in YBOCS score in P1. However, nonlinear dose dependency has been proposed as a principle of action in tDCS. Research on motor cortex stimulation has shown that previously postulated inhibiting effects of cathodal stimulation as well as exciting effects of anodal stimulation might be inversed depending on ground activity of stimulated tissue, current intensity and frequentness of stimulation (Brunoni et al., 2012; Batsikadze et al., 2013). It is likely that the impact of the stimulation parameters, i.e., current strength, polarity, duration and interval between stimulations, strictly depends on the stimulated area and might have led to counter regulatory or homeostatic plasticity effects in motor cortical areas as previously shown in neurophysiology studies (Nitsche and Paulus, 2001; Monte-Silva et al., 2010). Therefore, the mere transmission of those anodal stimulation parameters used to enhance dorsolateral prefrontal function in depressed patients to a hypothesized decrease of hyperactivity by cathodal motor cortex stimulation in GTS patients might induce unwanted non-linear, even inverse effects. Here, guidance on stimulation intensity, duration, and frequentness is still lacking and therapeutic options are restricted to empirical considerations. The issue of a critical dosage has been very recently emphasized in a rat model of GTS where different polarities and current intensities were applied (Edemann-Callesen et al., 2018). There, tDCS showed a polarity-specific (anodal) and non-linear intensity dependent (i.e., 200 μA as the only effective tDCS intensity) reduction of repetitive behavior in DAT-*tg* rats with hyperresponsive dopaminergic system in comparison to wildtype rats. The authors hypothesize that tDCS restores previously imbalanced sensorimotor striatal-thalamo-cortical networks in DAT-*tg* rats.

Finally, GTS is a complex disorder with highly variable phenotypic manifestations and high frequency of comorbidities (Qi et al., 2017). It is possible that the effects of tDCS treatment depend on a variety of genomic variants and chromosomal aberrations making a linear tDCS effect in the whole patient sample unlikely and showing the need for a further characterization of genetic subtypes and their impact on tDCS efficacy.

Thus, development of tDCS under use of translational research options rather than single-case guided research may represent an alternative and promising strategy towards development of tDCS as potential treatment for GTS. Finally, there may be a need to define tDCS intensities based on electrical field modeling than applying fixed tDCS intensities (Lisanby, 2017).

## Conclusion

Our case series shows that modulating cortical excitability in GTS has not yet been developed to a clinically applicable treatment. A proof-of-concept study in humans is still lacking. Due to the variety and complexity of cortical regions involved in GTS, there is a multitude of possible tDCS approaches but also interferences that inhibit prognoses of, or change expected results. Our results hint that future research will have to focus on the determination of most effective stimulation modes regarding site, polarity and frequentness in a first step before exploring tDCS in a larger clinical setting.

## Author Contributions

BL, EM, BK, LW, EW and LL helped with data preparation. NB and UP drafted the manuscript. FP, RM, BB and CW helped with proof reading.

## Conflict of Interest Statement

UP received paid speakership from neuroCare Group and has a private practice with neuroCare Group, Munich, Germany. FP received research support from NeuroConn GmbH, Ilmenau, Germany, and Brainsway Inc., Tel Aviv, Israel, as well as speaker’s honorarium from Mag & More GmbH, Munich, Germany, and neuroCare Group. LW is part-time employee of neuroCare Group. The remaining authors declare that the research was conducted in the absence of any commercial or financial relationships that could be construed as a potential conflict of interest.
